# Caffeine during High-Intensity Whole-Body Exercise: An Integrative Approach beyond the Central Nervous System

**DOI:** 10.3390/nu13082503

**Published:** 2021-07-22

**Authors:** Adriano E. Lima-Silva, Gislaine Cristina-Souza, Marcos D. Silva-Cavalcante, Romulo Bertuzzi, David J. Bishop

**Affiliations:** 1 Human Performance Research Group, Federal University of Technology Parana (UTFPR), Curitiba 81310900, PR, Brazil; limasilvaae@hotmail.com (A.E.L.-S.); gicsouzaufla@gmail.com (G.C.-S.); 2 Nutrition and Exercise Research Group, State University of Minas Gerais (UEMG), Passos 37902092, MG, Brazil; 3 Postgraduate Program in Nutrition (PPGNUT), Faculty of Nutrition (FANUT), Federal University of Alagoas (UFAL), Maceio 57072900, AL, Brazil; cavalcantemds@hotmail.com; 4 Endurance Sports Research Group (GEDAE-USP), University of São Paulo, Sao Paulo 05508030, SP, Brazil; bertuzzi@usp.br; 5 Institute for Health and Sport (IHES), Victoria University, Melbourne, VIC 8001, Australia

**Keywords:** methylxanthines, exercise performance, exercise-induced hypoxemia, muscle blood flow, central fatigue, peripheral fatigue

## Abstract

Caffeine is one of the most consumed ergogenic aids around the world. Many studies support the ergogenic effect of caffeine over a large spectrum of exercise types. While the stimulatory effect of caffeine on the central nervous system is the well-accepted mechanism explaining improvements in exercise performance during high-intensity whole-body exercise, in which other physiological systems such as pulmonary, cardiovascular, and muscular systems are maximally activated, a direct effect of caffeine on such systems cannot be ignored. A better understanding of the effects of caffeine on multiple physiological systems during high-intensity whole-body exercise might help to expand its use in different sporting contexts (e.g., competitions in different environments, such as altitude) or even assist the treatment of some diseases (e.g., chronic obstructive pulmonary disease). In the present narrative review, we explore the potential effects of caffeine on the pulmonary, cardiovascular, and muscular systems, and describe how such alterations may interact and thus contribute to the ergogenic effects of caffeine during high-intensity whole-body exercise. This integrative approach provides insights regarding how caffeine influences endurance performance and may drive further studies exploring its mechanisms of action in a broader perspective.

## 1. Introduction

**Figure 1:**
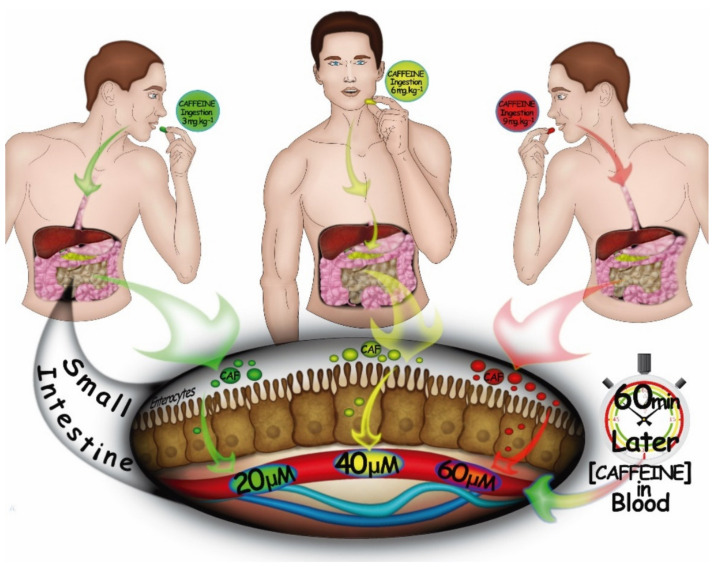
An illustration of the caffeine absorption process. Following doses of 3 to 9 mg kg^−1^ of body mass, caffeine is completely absorbed by the gastrointestinal tract and peaks in the plasma in a dose-dependent manner within approximately 60 min after ingestion [4].

**Figure 2:**
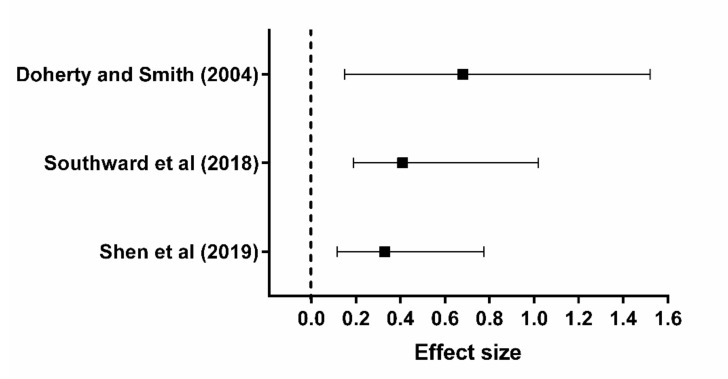
The ergogenic effect of caffeine ingestion on endurance performance measured using time trials (closed-loop exercises [9,11]), or time-to-task failure trials (open-loop exercises [12]).

Caffeine (1,3,7-trimethylxanthine) is an alkaloid widely used to improve exercise performance [1,2]. Following doses of 3 to 9 mg kg^−1^ of body mass, caffeine is completely absorbed by the gastrointestinal tract and peaks in the plasma (20 to 60 µM, in a dose-dependent manner) within approximately 1 h after ingestion [3,4] (Figure 1). Caffeine can pass through all biological membranes and is readily distributed throughout all tissues of the body [5]. Its subsequent ergogenic effects have been demonstrated for many types of exercise, such as those involving muscle endurance, muscle strength, anaerobic power, and aerobic endurance [6,7,8]. Regarding aerobic endurance, caffeine seems to improve performance in both closed-loop exercises, in which work rate can be continually regulated in an attempt to complete the task as quickly as possible, and in open-loop exercise, in which work rate is fixed and exercise is performed to the limit of exhaustion [9,10,11,12] (Figure 2).

An important characteristic of aerobic endurance is that it typically requires the recruitment of a large muscle mass. Thus, “whole-body exercise” (e.g., cycling and running) is of special interest because of its functional relevance and significant engagement and interaction of various physiological systems [13]. The second characteristic of aerobic endurance is that exercise encompasses a large range of exercise durations (from 75 seconds to hours, see Gastin [14]). Furthermore, the stress imposed on various physiological systems during whole-body endurance exercise will increase as a function of exercise intensity. Specifically, endurance exercise performed above critical power or 85 to 90% of maximal oxygen uptake ($\dot{V}$O_2_max), which can be defined as “severe-intensity exercise” or “high-intensity exercise”, is associated with progressive derangements of homeostasis and rapid achievement of $\dot{V}$O_2_max, with subsequent attainment of task failure [15]. It is noteworthy that caffeine retains its ergogenic effects even in endurance exercise with such characteristics [16].

Caffeine-induced improvements in endurance performance are commonly associated with mechanisms residing within the central nervous system, via its inhibitory action on A_1_ and A_2a_ adenosine receptors [3]. However, the abundance of adenosine A_1_ and A_2a_ receptors in other non-nervous tissues (e.g., heart, blood vessels, lungs, and skeletal muscle [17,18]), and the special characteristic of high-intensity whole-body exercise, in which several physiological systems are stressed to their limit of tolerance, suggest it is important to assess the potential effects of caffeine on other physiological systems during this mode of exercise [19]. An understanding of the effects of caffeine on multiple physiological systems during whole-body, high-intensity exercise might help expand its use in different sporting contexts or even for the treatment of some diseases. For example, a potential effect of caffeine influencing the pulmonary system and maintaining arterial O_2_ saturation (S_a_O_2_) might be helpful for athletes competing in hypoxic environments at high altitude or for patients with chronic obstructive pulmonary disease engaging in an exercise training program [20,21]. Therefore, in the present narrative review, we provide a comprehensive overview of the potential effects of caffeine on the pulmonary, cardiovascular, and skeletal muscle systems, and how such effects might connect with the CNS during high-intensity whole-body exercise. We also present an integrative approach, providing some insights about the different pathways by which caffeine might influence endurance performance during high-intensity whole-body exercise. The terms and Boolean operators “caffeine” AND “pulmonary system” OR “cardiovascular system” OR “muscular system” OR “peripheral fatigue” OR “central fatigue” were used in the search strategy in the PubMed/MEDLINE, Scopus, Web of Science and Science Direct databases.

## 2. The Pulmonary System

The primary objective of the pulmonary system during exercise is to maintain the partial pressures of oxygen and carbon dioxide in the blood (PO_2_ and PCO_2_, respectively) as close as possible to resting values [13,22]. To meet this requirement, respiratory muscle work and alveolar ventilation increase considerably during high-intensity whole-body exercise [23]. The capacity of the pulmonary system is generally assumed as sufficient to match the demands imposed by endurance exercise [22]. However, there is research indicating that the pulmonary demands associated with high-intensity whole-body exercise may outstrip the functional capacity of the respiratory system and eventually compromise arterial O_2_ content [13,22,23]. In general, the three main limitations associated with the pulmonary system response to high-intensity whole-body exercise are: (1) exercise-induced arterial hypoxemia, (2) respiratory muscle fatigue, and (3) expiratory positive pressure effects on cardiac output.

Exercise-induced arterial hypoxemia refers to a reduction in S_a_O_2_ greater than 5% from resting levels (S_a_O_2_ is ~98% at rest) [22]. Although some studies suggest a similar phenomenon during less intense exercise [24,25], exercise-induced arterial hypoxemia is commonly reported during sustained high-intensity whole-body exercise and may compromise exercise tolerance [24,25,26]. A detailed discussion about the main mechanisms governing exercise-induced arterial hypoxemia is beyond the scope of this review and readers are directed to other works [21,23,27]. In brief, exercise-induced arterial hypoxemia has been attributed to multiple mechanisms such as inadequate compensatory hyperventilation, excessive widening of the alveolar to arterial O_2_ difference, and a rightward shift of the oxyhemoglobin dissociation curve (acid- and temperature-induced). Regardless of the mechanisms, exercise-induced arterial hypoxemia might compromise O_2_ transport to active muscles [28,29].

Fatigue of the respiratory muscles can also compromise O_2_ transport to active muscles. Accumulation of metabolites associated with fatiguing contractions of the inspiratory and expiratory muscles activate metabosensitive phrenic nerves, which may reflexively increase sympathetic vasoconstriction of the vasculature in locomotor muscles and ultimately reduce muscle perfusion and O_2_ delivery in an attempt to preserve/increase blood flow to the respiratory muscles [28,30]. In addition, increases in positive expiratory intrathoracic pressure, not sufficiently counterbalanced by a reduction in negative inspiratory intrathoracic pressure, will increase the ventricular afterload and thereby decrease stroke volume during high-intensity whole-body exercise [22]. Such alterations might reduce the blood flow to the locomotor muscles, reducing O_2_ supply to the active muscles.

A question arising from the above is whether caffeine ingestion can attenuate the imposed respiratory system limitations during high-intensity whole-body exercise. There is some research suggesting that caffeine influences ventilation ($\dot{V}$E), end-tidal O_2_ partial pressure (P_ET_O_2_), and S_a_O_2_ during progressive treadmill exercise to exhaustion in athletes with moderate-to-severe exercise-induced hypoxemia [20]. Specifically, caffeine increased P_ET_O_2_ and S_a_O_2_ during the highest workloads (75 to 90% of $\dot{V}$O_2_max), which was associated with an increased $\dot{V}$E. Interestingly, caffeine increased $\dot{V}$E at 100% of $\dot{V}$O_2_max but this was not enough to counteract the reduction in S_a_O_2_ with the increase in workload [31]. It was suggested that a large portion of the increased $\dot{V}$E with caffeine at this exercise intensity may have reached dead space ventilation rather than alveolar ventilation.

The mechanisms by which caffeine increases $\dot{V}$E and prevents arterial O_2_ desaturation during high-intensity whole-body exercise are not fully known. Studies in resting animals [32] and humans [33] suggest a centrally-mediated effect of caffeine, where caffeine may directly act on the respiratory center and increase its sensitivity to carbon dioxide. Equivocal results, however, have been reported about the action of caffeine on peripheral chemoreceptors. While caffeine seems to increase the resting ventilatory response to progressive isocapnic hypoxia and hyperoxic hypercapnia in healthy men [34], a study with well-trained athletes failed to find such effects [31]. Whether such central and/or peripheral effects of caffeine are preserved during high-intensity whole-body exercise is difficult to experimentally assess. Caffeine also promotes relaxation of smooth muscles of the bronchi, inducing bronchodilatation and protecting from bronchoconstriction induced by a bronchoprovocation challenge with dry gas or exercise [35,36]. As near-maximal bronchodilatation probably already occurs during high-intensity whole-body exercise [37,38], it is not known whether this caffeine-induced bronchodilation effect will remain important in such conditions. Finally, caffeine augments the contractility and endurance of the inspiratory muscles, while concomitantly reducing the sense of effort associated with fatiguing inspiratory muscle contractions, probably by overcoming a fatigue-induced reduction in the outflow from the central respiratory motoneuron pool and/or altering respiratory motor neuron firing patterns [39].

**Figure 3:**
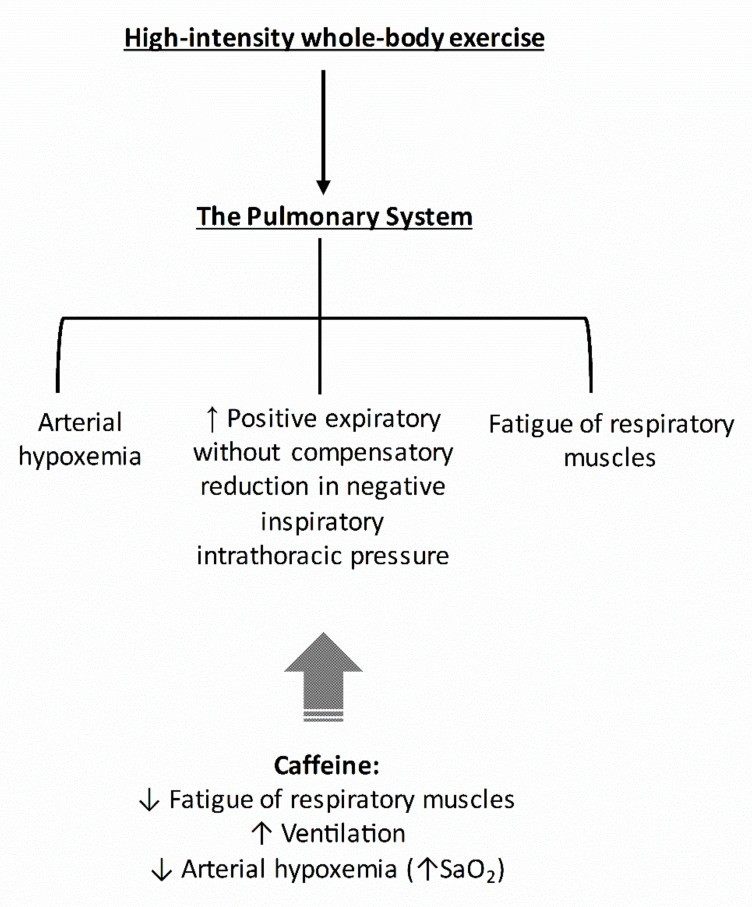
Main physiological events within the pulmonary system that might contribute to fatigue during high-intensity whole-body exercise. The potential influence of caffeine on components of the pulmonary system that might explain part of its ergogenic effect is also indicated. Caffeine reduces fatigue of respiratory muscles [39] and increases pulmonary ventilation and arterial oxygen saturation [20,31].

As the pulmonary system imposes considerable limitations during high-intensity whole-body exercise, the caffeine-induced increases in $\dot{V}$E and the preservation of near resting S_a_O_2_ may account for part of the ergogenic effects of caffeine in this mode of exercise. In Figure 3, we provided a hypothetical construct by which caffeine might affect the pulmonary system.

## 3. The Cardiovascular System

The cardiovascular system provides the link between pulmonary ventilation and oxygen usage at the cellular level. During high-intensity whole-body exercise, heart rate and stroke volume (and consequently cardiac output, $\dot{Q}$) increase progressively until reaching maximal values [40]. Leg blood flow ($\dot{Q}$_L_) will also be close to maximal and might limit both muscle O_2_ delivery and endurance exercise performance [28]. As written earlier, although controversial [41], $\dot{Q}$_L_ during high-intensity whole-body exercise may be reflexively reduced via vasoconstriction of the vasculature of the exercising limb due to the increased O_2_ demand by the respiratory musculature [28]. In addition, a substantial augmentation of expiratory positive pressures during strenuous exercise may decrease stroke volume, further exacerbating the limitation of $\dot{Q}$_L_ [42].

Data related to the effects of caffeine ingestion on stroke volume, $\dot{Q}$, and $\dot{Q}$_L_ during high-intensity whole-body exercise are limited. To our knowledge, only one study has investigated this question and found no increase in stroke volume or $\dot{Q}$ during an incremental, symptom-limited, maximal supine bicycle exercise after caffeine ingestion [43]. There are more data regarding the effects of caffeine on maximal heart rate, but results are contradictory with some studies showing increased maximal heart rate [44,45] and others not [46,47,48]. However, most studies showing no effect of caffeine on maximal heart rate were conducted using multiple, 60-s, maximal cycling bouts [46,47], which may not have allowed sufficient time to reach maximal heart rate. Studies showing a positive effect of caffeine on maximal heart rate were performed using a maximal incremental test [44,45]. Indeed, using a constant-load, high-intensity whole-body exercise performed until volitional exhaustion, it was demonstrated that caffeine increased end-exercise heart rate by ~3% [16]. Whether this marginal effect on heart rate will be enough to affect $\dot{Q}$_L_ is not known, but as stroke volume might plateau at about 40% of $\dot{V}$O_2_max with no further increase despite increasing intensity of exercise [49], a caffeine-induced increase in end-exercise heart rate during strenuous exercise might result in a small increase in $\dot{Q}$. Further research will be necessary to test this assumption.

**Figure 4:**
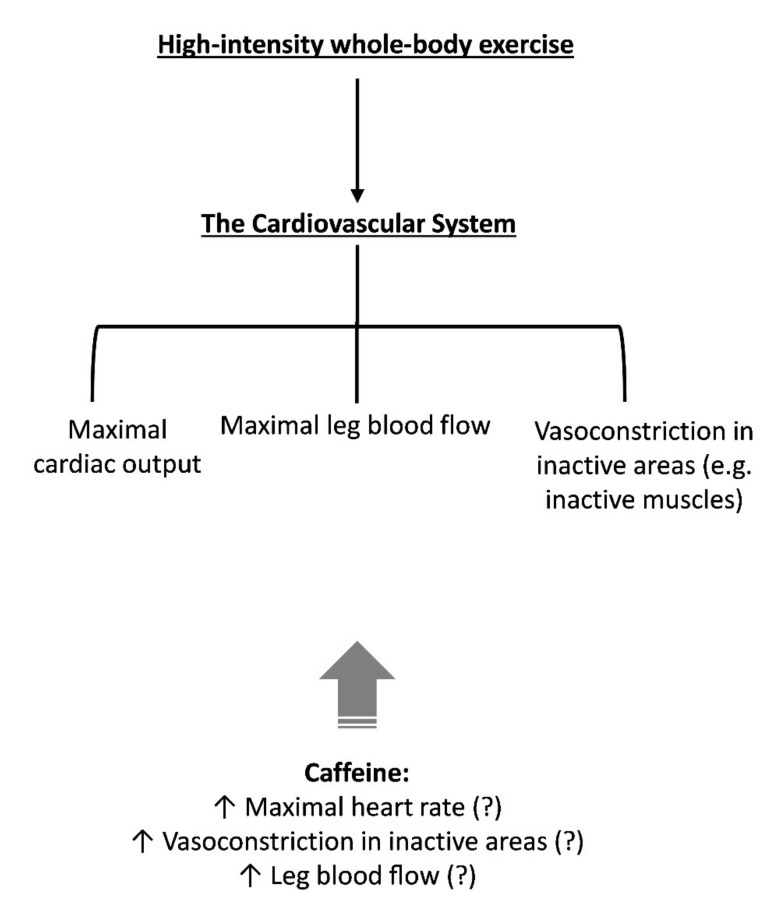
Main physiological events within the cardiovascular system that might contribute to fatigue during high-intensity whole-body exercise. The potential influence of caffeine on components of the cardiovascular system that might explain part of its ergogenic effect is also indicated. Although inconclusive, caffeine might increase maximal heart rate [44,45] and vasoconstriction in inactive areas [43,53], which may result in increased leg blood flow.

As $\dot{Q}$ will be close to maximal during high-intensity whole-body exercise, several systems will be competing for the redistribution of this “limited” $\dot{Q}$. Many factors can influence the redistribution of blood flow during exercise, such as active muscle mass [50,51] and respiratory muscle work [30]. Furthermore, the well-known adenosine-receptor antagonism of caffeine may also impact blood flow redistribution. Indeed, adenosine causes vasodilation in several regional circulations and attenuates the release of renin [52]; therefore, blockage of adenosine receptors in blood vessels and the kidney via caffeine might alter blood flow redistribution. Although no data exist related to high-intensity whole-body exercise, it was demonstrated that caffeine acts as a powerful vasoconstrictor of inactive regions during dynamic leg exercise (cycle ergometry) at moderate intensity (65% $\dot{V}$O_2_max), increasing plasma angiotensin II (a powerful vasoconstrictor) and attenuating forearm blood flow (−53%) and forearm vascular conductance (−50%), which results in an increased mean arterial blood pressure [53]. Extrapolation of these results to near-maximal exercise should be done with caution, but some evidence suggests caffeine exacerbates the blood pressure response and plasma epinephrine concentration (another vasoconstrictor) during maximal exercise [43]. In Figure 4, we provided a hypothetical construct by which caffeine might affect the cardiovascular system. Further studies, however, will be necessary to investigate whether caffeine can increase $\dot{Q}$_L_ during high-intensity whole-body exercise.

## 4. The Skeletal Muscle

Locomotor muscle oxygen uptake is a function of the product of $\dot{Q}$_L_ and arterial-mixed venous oxygen difference (the Fick principle). The reduction in O_2_ delivery to active muscles by O_2_ desaturation and/or reduced blood flow may constrain high-intensity whole-body exercise performance by accelerating locomotor muscle fatigue [26]. During a high-intensity whole-body exercise carried out at identical work rates and for equal durations, preventing the normal O_2_ desaturation incurred during exercise reduces by about one-half the amount of exercise-induced quadriceps’ fatigue (as measured by the pre- to post-exercise reduction in evoked twitch force by supramaximal electrical or magnetic peripheral motor nerve stimulation) [26]. When S_a_O_2_ is “clamped” at 85% (moderate hypoxemia), locomotor muscle fatigue development over exercise time is exacerbated [54].

Recent findings suggest that caffeine increases active muscle oxygen saturation (measured via near-infrared spectrometer, NIRS) during submaximal workloads (30 to 60% of $\dot{V}$O_2_max) during a maximal incremental test with 1-min steps [48]. However, caffeine failed to increase muscle saturation at the highest workloads. It is noteworthy that only a main effect of substance was found. An absence of substance vs. workload interaction prevents a separate analysis for each workload without inflating type I error. A more suitable conclusion would be that caffeine increases muscle oxygen saturation during all workloads. In addition, it is important to consider that muscle oxygen saturation measured via NIRS-derived signals reflect the relationship between local muscle O_2_ delivery and muscle O_2_ utilization within the region of NIRS interrogation; thus, it is not possible to affirm from this data whether caffeine increased blood flow and consequently O_2_ delivery to the active muscles.

Despite the scarcity of studies investigating the effect of caffeine on $\dot{Q}$_L_, there is accumulating evidence using single-leg knee extensor exercise that caffeine attenuates locomotor muscle fatigue development [55,56]. While a direct relationship between increased O_2_ delivery and a reduction in locomotor muscle fatigue cannot be concluded from current data available in the literature, a direct effect of caffeine on skeletal muscles may also explain a reduced rate of peripheral fatigue development. It is, however, difficult to experimentally isolate a direct effect of caffeine on skeletal muscle in humans. In an attempt to isolate the muscular effects of caffeine, a study with electrical stimulation of the ulnar nerve (i.e., without CNS influence) showed an increase in muscle tension after caffeine ingestion before and after a protocol of muscle fatigue in healthy adults [57]. Caffeine also increased exercise tolerance in a group of spinal-cord-injured men during functional electrical stimulation of their paralyzed limbs to the point of fatigue [58]. Another study showed that caffeine potentiated the force of contraction during the final minute of a 2-min tetanic stimulation of the common peroneal nerve in healthy men [59]. However, a recent study failed to find an effect of caffeine ingestion on evoked forces in fresh quadricep muscles of healthy men [60]. Together, the results of these studies using electrical stimulation techniques argue in favor of a potential effect of caffeine on skeletal muscles, but its action seems to be more evident in studies in exercised/fatigued [58,59,61,62,63] than fresh muscles [60,64].

Studies investigating the effects of caffeine on muscle contractility of isolated mouse muscle fibers also provide important insights regarding caffeine’s effect on skeletal muscle. A recent study reported that toxic doses of caffeine (>1000 µM) were required to increase electrically-stimulated force in intact single fibers of mouse flexor digitorum brevis muscle [60]. Many studies, however, have demonstrated that mouse soleus muscle placed in a muscle rig with a circulating solution containing 70 µM of caffeine (near-maximum, non-toxic plasma concentration attainable in humans) increases its power output during 60 min of electrical stimulation [61,62,63]. As a direct effect of caffeine on muscle contractility of isolated mouse muscle fibers was evident on exercised [61,62,63] but not resting muscle [60], these findings are in agreement with findings using electrically-stimulated muscle in humans in which caffeine increased muscle force in exercised [57,58,59] but not fresh muscle [60]. Another important finding is that caffeine increased muscle contractility with a dosage (~70 µM) close to the limit of tolerance for plasma caffeine concentration in humans [61,62,63]. It has been reported that caffeine ingestion at doses of 9 mg kg^−1^ increased plasma caffeine concentration to ~70 µM one hour after ingestion in adult men [4]. Therefore, a direct effect of caffeine on muscle contractility can be obtained using non-toxic plasma caffeine concentrations for humans.

Caffeine-induced improvements in muscle function have mainly been attributed to the caffeine antagonism of adenosine A_1_ receptors on the skeletal muscle membrane, and/or by direct binding to ryanodine receptors resulting in greater Ca^2+^ release from the sarcoplasmic reticulum, and/or increased myofibrillar Ca^2+^ sensitivity [61]. A study using fresh muscle and micromolar physiological concentrations of caffeine has refuted this Ca^2+^-related mechanism [60], but experimental data during exercised/fatigued muscle is lacking. The direct effect of caffeine on muscle should be more evident in the later stages of fatigue when the contractile machinery in muscle fibers is not fully activated due to reduced sarcoplasmic reticulum Ca^2+^ release and decreased myofibrillar Ca^2+^ sensitivity. Caffeine can also reduce both plasma [65] and muscle interstitial K^+^ [66], which is compatible with a caffeine-induced increase in muscle fiber membrane excitability (as measured by peak-to-peak M_wave_ amplitude) [64]. Some evidence also suggests that individuals responsive to caffeine have attenuated muscle phosphocreatine degradation and reduced intramuscular accumulation of free ADP and free AMP after 3 min of a high-intensity (80% $\dot{V}$O_2_max) exercise [67]. Together, these potential peripheral sites of action of caffeine suggest that caffeine ingestion might improve the metabolic milieu in skeletal muscle during exercise, which may account for part of its ergogenic effect.

**Figure 5:**
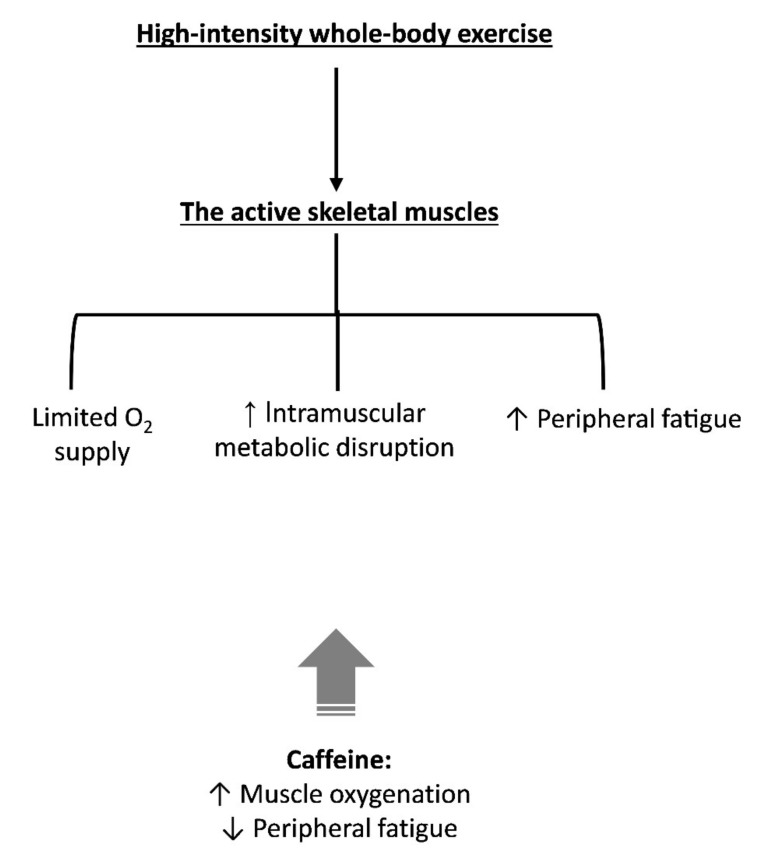
Main physiological events within the skeletal muscle system that might contribute to fatigue during high-intensity whole-body exercise. The potential influence of caffeine on components of the skeletal muscle system that might explain part of its ergogenic effect is also indicated. Caffeine might increase muscle oxygenation [48] and the capacity of skeletal muscle in generating force when fatigued, i.e., reduce peripheral fatigue [57,58,59,61,62,63].

In summary, a reduction in exercise-induced locomotor fatigue is documented after caffeine ingestion, which could be a consequence of an improved O_2_ delivery [48] and/or associated with a direct effect of caffeine on skeletal muscle [57,58,59,61,62,63]. Such alterations might result in an improved intramuscular metabolic milieu. In Figure 5, we provided a hypothetical construct by which caffeine might affect skeletal muscle.

## 5. Connection between Peripheral and Central Nervous Systems

If caffeine improves the metabolic milieu in skeletal muscle, it would be expected that the inhibitory feedback to the CNS may also be reduced. Metabosensitive and mechanosensitive group III/IV locomotor muscle afferents project to cortical and spinal regions of the CNS [68,69]. The role of these muscle afferents in limiting locomotor muscle fatigue has been demonstrated by a series of experiments pharmacologically blocking the central projection of these sensory neurons [70,71,72], and readers are directed to excellent reviews about this topic [13,73,74]. Briefly, during high-intensity whole-body exercise, the central projection of group III/IV muscle afferent feedback constrains central motor drive to active muscles and limits the exercise-induced intramuscular metabolic perturbation, so as to protect locomotor muscles from severe intramuscular metabolic disturbance [70]. Thus, the peripheral effects of caffeine might attenuate the afferent signaling from working muscles to the CNS, reducing the inhibitory influence of fatiguing locomotor muscle fatigue on central motor drive [64]. In addition, adenosine A_2a_ receptor activation sensitizes peripheral afferent fibers that project to the spinal cord, enhancing nociception [75]. Caffeine, via its antagonistic actions on the A_2a_ receptor, may attenuate pronociceptive actions of adenosine at the spinal level [76]. This caffeine-induced attenuation in both metabolic and nociceptive afferent signals may be centrally integrated and perhaps summed to achieve the well-recognized central effects of caffeine in the CNS.

As traditionally accepted, there is, however, no doubt that caffeine also acts directly on the CNS to ultimately influence efferent pathways [77,78,79,80,81]. A study quantifying the in vivo occupancy of the human cerebral A_1_ adenosine receptors by caffeine, using 18F-CPFPX and PET techniques, showed that a plasma caffeine concentration of ~70 µM blocked 50% of the cerebral A_1_ receptors [82]. As a consequence, it has been demonstrated using the transcranial magnetic stimulation technique that caffeine decreases the cortical silent period [83] and increases corticospinal excitability [77,78]. In addition, the potentials evoked by cervicomedullary stimulation of the descending corticospinal tract are also increased with caffeine, suggesting an increased excitability of the corticospinal neurons [79]. Finally, caffeine increases the slope of the H-reflex recruitment curve, reflecting an increased spinal excitability [80].

An important result of the attenuated afferent signals combined with the increased cortical and spinal excitability provoked by caffeine ingestion is a change in muscle activation pattern during exercise. When participants are free to adjust their exercise intensity, as during closed-loop exercise (e.g., a 4-km cycling time trial), caffeine increases *vastus lateralis* activation (quantified via electromyography signal), suggesting increased central motor drive to active muscles [64]. Interestingly, the magnitude of increase in muscle activation after caffeine ingestion is considerably lower than that observed with fentanyl injection impairing cortical projection of opioid-mediated muscle afferents [72], suggesting that caffeine attenuates but not fully blocks afferent signals to the CNS, which allows proper pacing and optimizing of exercise performance [64]. In addition, caffeine also attenuates the rate of decline in voluntary muscle activation measured via the twitch-interpolation technique [56], an index of the capacity of the CNS to maximally activate the working muscles [13,84]. A reduction in the capacity of the CNS to maximally activate the working muscles is assumed to represent a “central fatigue”, which means that processes causing fatigue are residing within the CNS [13,84]. Facilitated central motor drive caused by the reduced afferent signals and/or increased excitability of the motor pathways may contribute to a reduction in both central and peripheral fatigue after caffeine ingestion.

## 6. An Integrative Approach to Caffeine Effects during High-Intensity Whole-Body Exercise

**Figure 6:**
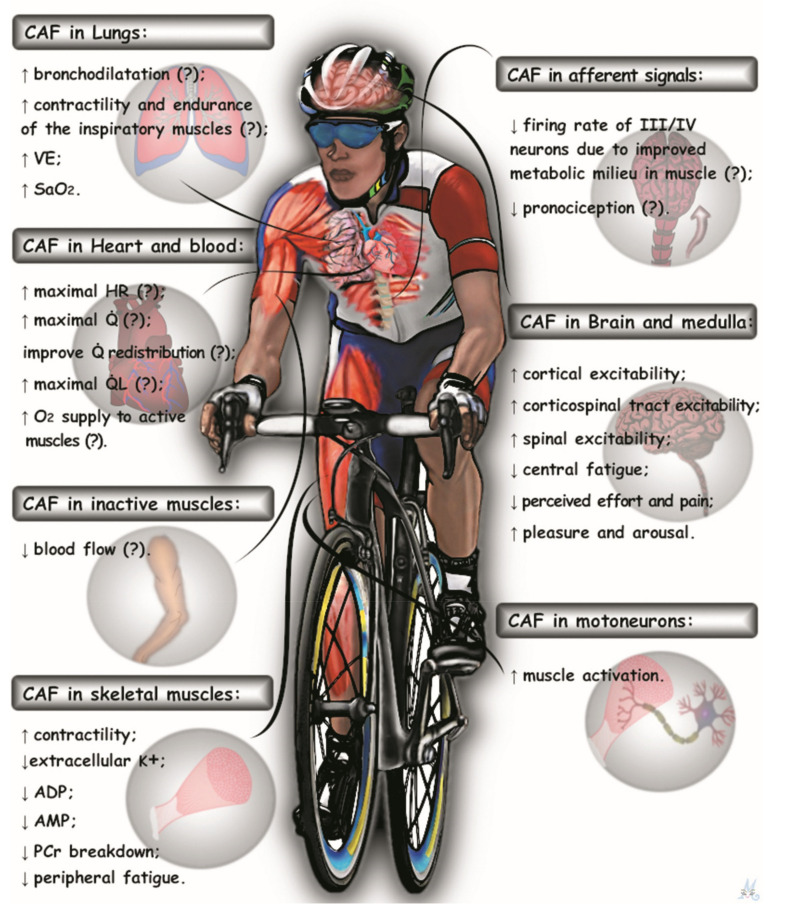
An illustration of the integrative approach to the effects of caffeine (CAF) on multiple physiological systems. Together, such alterations may better explain the improvements in endurance performance during high-intensity whole-body exercise. $\dot{V}$E: pulmonary ventilation. S_a_O_2_: arterial oxygen saturation. HR: heart rate. $\dot{Q}$: cardiac output. $\dot{Q}$_L_: leg blood flow. K^+^: extracellular potassium. ADP: adenosine diphosphate. AMP: adenosine monophosphate. PCr: phosphocreatine.

Evidence for positive effects of caffeine during high-intensity whole-body exercise beyond mechanisms attributed solely to the CNS is now accumulating. Figure 6 provides a summary of a potential integrative picture of the effects of caffeine on multiple physiological systems.

This hypothetical construct might assist further research investigating the effect of caffeine during high-intensity whole-body exercise. Caffeine might affect the maintenance of blood oxygenation and blood flow redistribution to active muscles, which may increase muscle oxygenation that, associated with a direct effect of increasing contractility in skeletal muscle, would imply a reduced rate of exercise-induced locomotor muscle fatigue. Thus, further studies investigating the effect of caffeine during high-intensity whole-body exercise should measure blood flow and O_2_ delivery to active and inactive muscles. In addition, exercise-induced locomotor muscle fatigue should also be measured. A reduced rate of peripheral fatigue development will reduce the inhibitory feedback from fatiguing muscle to the central motor drive. Together with increased cortical/spinal/muscle excitability, improved muscle recruitment patterns will preserve the ability of the CNS to maximally activate the working muscles. This improved physiological environment may also explain some perceptive alterations during high-intensity exercise after caffeine ingestion, such as a reduction in perceived effort and pain, and increased sensation of pleasure and arousal [85,86,87]. This integrative perspective might better explain the caffeine-induced improvements in endurance performance during high-intensity whole-body exercise than exclusively attributing ergogenic effects to a “central effect”. We noted, however, that there are limited published data available for some points of this hypothetical construct (e.g., cardiac output, blood flow redistribution, muscle blood flow, and O_2_ delivery). Thus, further studies measuring different parameters of the cardiopulmonary, cardiovascular, and muscular systems will be necessary to provide further support for our proposed explanation of the multiple effects of caffeine during high-intensity whole-body exercise.

## 7. Conclusions

Caffeine is a recognized ergogenic aid and its effect on endurance performance is well accepted. However, the underlying mechanisms responsible for the ergogenic effects of caffeine remain intriguing as several mechanisms residing outside the CNS have been underexplored in the literature. In the present review, we have provided some insights into how caffeine might directly act on the pulmonary, cardiovascular, and muscular systems, which may contribute to an integrative perspective on the ergogenic effects of caffeine. Further studies investigating the mechanisms associated with caffeine-induced improvements in performance during high-intensity whole-body exercise may benefit from considering this integrative approach.

## Data Availability

Not applicable.
